# Co-Occurrence of an NDM-1-Carrying SGI1 Variant and a Novel GI*flu*-1 Resistance Island in a Seafood-Derived *Vibrio fluvialis*

**DOI:** 10.3390/vetsci13070639

**Published:** 2026-06-30

**Authors:** Ming Liu, Wenhui Zhang, Wanyu Zhang, Patrick Butaye, Zhiqiang Wang, Ruichao Li

**Affiliations:** 1 College of Nursing and Health Management, Wuhan Donghu University, Wuhan 430040, China; 20190018@hhu.edu.cn; 2 Institute of Comparative Medicine, Yangzhou University, Yangzhou 225012, China; wenhuizhang_yzu@outlook.com (W.Z.); awan46110@163.com (W.Z.); zqwang@yzu.edu.cn (Z.W.); 3 Jiangsu Co-Innovation Center for Prevention and Control of Important Animal Infectious Diseases and Zoonoses, College of Veterinary Medicine, Yangzhou University, Yangzhou 225012, China; 4 Department of Infectious Diseases and Public Health, Jockey Club College of Veterinary, City University of Hong Kong, Hong Kong SAR, China; 5 Faculty of Veterinary Medicine, Department of Pathobiology, Pharmacology and Zoological Medicine, Ghent University, 9000 Merelbeke, Belgium

**Keywords:** *Vibrio fluvialis*, NDM-1, SGI1, resistance, One Health

## Abstract

*Vibrio fluvialis* is an important zoonotic pathogen that poses a growing threat to both public health and aquaculture, particularly to shrimp and fish farming. In this study, we report that a seafood-associated *V. fluvialis* strain, 10M-VF, possesses two genomic islands, SGI1-VfNDM1 and GI*flu*-1, in addition to hemolysins and type VI secretion systems (T6SSs). SGI1-VfNDM1 is a new member of the *Salmonella* genomic island 1 (SGI1) and harbors several antibiotic resistance genes. A potential transferable region, IS*CR1*-*trpF*-*ble*_MBL_-*bla*_NDM-1_, was observed in SGI1-VfNDM1, suggesting that IS*CR1* may have mediated the integration of *bla*_NDM-1_ into this island. Additionally, GI*flu*-1 represents a novel resistance island. Other GI*flu* variants carrying resistance genes in *Vibrio* all possess IS*CR2*, but their resistance regions vary. This indicates that IS*CR2* may contribute to the integration of antibiotic resistance genes into GI*flu* islands. Notably, not every GI*flu* variant carries drug resistance genes. To the best of our knowledge, GI*flu*-1 is a previously unreported resistance island, and no previous report has documented the co-existence of two distinct resistance genomic islands in a single *Vibrio* isolate. The emergence of such resistance islands in a seafood-borne pathogen raises concerns for the maintenance and potential dissemination of clinically important resistance genes in aquatic food systems and underscores the need for ongoing surveillance of resistance determinants in seafood-borne *Vibrio* populations from a One Health perspective.

## Simple Summary

Carbapenems are critically important antibiotics for treating severe bacterial infections in humans and animals. A carbapenem-resistant *V. fluvialis* strain was identified from retail mantis shrimp in this study. The presence of two different genomic islands carrying multiple antibiotic resistance genes in a single seafood-borne strain is particularly concerning, as it suggests that such bacteria can serve as reservoirs for resistance genes that could spread to other bacteria in the aquatic environment and potentially affect food safety. These findings highlight the importance of monitoring antibiotic resistance in *Vibrio* species from seafood to protect both animal and public health.

## 1. Introduction

*Vibrio fluvialis* is an important zoonotic pathogen. It is the causative agent of vibriosis in aquaculture and is responsible for significant economic losses in shrimp, lobster, and fish farming [1,2,3]. In China, this pathogen causes a substantial number of diarrhea cases every year, especially among individuals aged 5–17 years [4]. In India, this marine pathogen is also associated with many gastrointestinal diseases [5,6]. Given its economic importance and public health impact, the molecular mechanisms underlying *V. fluvialis* pathogenesis have been studied. This bacterium produces two hemolysins, VFH and δVFH. δVFH has been shown to induce pyroptosis in vitro [7]. VFH is a key virulence factor that induces severe colonic histopathological lesions in mouse models [8]. The type VI secretion system (T6SS) in *Vibrio* species is involved in interbacterial competition and the infection of eukaryotic host cells. Depending on the strain, *V. fluvialis* encodes one or two T6SSs. T6SS1 is suggested to have been horizontally acquired by some *V. fluvialis* strains, but its role remains unclear, possibly due to mutations in its structural genes that hinder functional studies [9]. In contrast, T6SS2 provides a competitive advantage in vitro [10]. The roles of these two hemolysins and T6SSs in the pathogenicity of *V. fluvialis* to aquatic animals, however, remain largely unexplored. The strain characterized in this study, 10M-VF, was also examined for the presence of these virulence determinants.

NDM (New Delhi metallo-β-lactamase) carbapenemases confer resistance to carbapenems. Several *Vibrio* species have been reported to produce NDM-1. For example, *V. fluvialis* strains isolated from both clinical settings and shrimp carry conjugative plasmids harboring *bla*_NDM-1_ [6,11]. Indeed, in *Vibrio* species, *bla*_NDM-1_ is predominantly located on plasmids [12,13]. However, alternative mobile elements, such as *Salmonella* genomic island 1 (SGI1) and its variants, also play a crucial role in the dissemination of antibiotic resistance genes among *Gammaproteobacteria*, and require an IncC helper plasmid for conjugative transfer [14]. Although SGI1 has been detected in some *Vibrio* species [14], the carriage of *bla*_NDM-1_ on SGI1 in *Vibrio* remains rare. To date, only one *V. fluvialis* strain from retail clam and four *V. cholerae* strains (one from blood, and other three of unknown origin) have been reported to carry *bla*_NDM-1_ on SGI1 [14,15].

Besides SGI1, several genomic islands have been implicated in the dissemination of antibiotic resistance genes in Gram-negative bacteria. Among them, GI*sul2* is a well-characterized integrative element carrying the sulfonamide resistance gene *sul2*, often associated with the IS*CR2* element. GI*sul2* typically resides in the chromosome or on plasmids and has been identified in various *Proteobacteria*, including *Enterobacter cloacae*, *Shigella flexneri*, and *Acinetobacter baumannii* [16,17]. The integrase of GI*sul2* mediates the excision and integration of both GI*sul2* and the CR2-*sul2* unit through site-specific recombination at *att* sites [17]. However, whether additional, previously unrecognized genomic islands exist in *Vibrio* species, or the coexistence of two distinct resistance genomic islands in a single *Vibrio* isolate, remains largely unknown.

*Oratosquilla oratoria* is a popular seafood in China, with an annual catch exceeding 200,000 tons, reflecting its high market demand and consumption [18]. Given the large-scale consumption, the potential for human exposure to *Vibrio* species through seafood is substantial. Seafood serves as a critical reservoir for *Vibrio*; therefore, genomic surveillance of seafood-borne isolates can uncover novel resistance determinants. In aquaculture, *Vibrio* infections are often treated with antibiotics, and the emergence of carbapenemase genes in *Vibrio* species could exacerbate treatment challenges, as carbapenems are not used in aquaculture but resistance genes can be maintained and potentially transferred to other bacteria in the aquatic environment. From a veterinary perspective, the presence of resistance genes in a seafood-borne pathogen highlights the need for integrated surveillance of resistance determinants in aquatic food systems. While a *bla*_NDM-1_-carrying SGI1 variant has been previously reported in a seafood-derived *V. fluvialis* strain [15], the key novelty of this study lies in the co-occurrence of a novel resistance island (GI*flu*-1) and an SGI1 variant (SGI1-VfNDM1) within a single isolate. The aim of this study was to elucidate the genetic basis of carbapenem resistance, identify associated mobile genetic elements, and characterize virulence determinants in *V. fluvialis* strain 10M-VF through whole-genome sequencing, and to assess the transferability of the identified resistance elements and their One Health implications for food safety and animal health.

## 2. Materials and Methods

### 2.1. Strain Isolation and Identification

In a surveillance study of carbapenemase-producing *Vibrio* species, retail seafood, including shrimp, shellfish, and mantis shrimp (*Oratosquilla oratoria*) [18] were collected from 6 different supermarkets in Nanjing, China, in March 2021. A total of 38 seafood samples were collected using a convenience sampling strategy. Each seafood sample (approximately 25 g) was homogenized in 50 mL alkaline peptone water (Hopebio, Qingdao, China) containing 1 μg/mL meropenem (Sigma-Aldrich, St. Louis, MO, USA) and incubated at 37 °C for 6 h under aerobic conditions with shaking at 180 rpm. The enrichment culture was then streaked onto thiosulfate–citrate–bile salts–sucrose (TCBS) agar (Hopebio, Qingdao, China) supplemented with 1 μg/mL meropenem. For each sample, the enrichment culture was streaked onto TCBS agar, and 2–3 colonies per plate were randomly selected for purification [19]. A single colony was purified and designated as strain 10M-VF. Species identification was confirmed by matrix-assisted laser desorption ionization-time of flight mass spectrometry (MALDI-TOF MS, Bruker Daltonics, Billerica, MA, USA) and multilocus sequence typing (MLST) using the PubMLST database (https://pubmlst.org/, accessed 18 May 2023).

### 2.2. Antimicrobial Susceptibility Testing and Conjugation Assay

Minimum inhibitory concentrations (MICs) for 13 antimicrobial agents (ampicillin, cefotaxime, ceftazidime, meropenem, imipenem, tetracycline, ciprofloxacin, gentamicin, sulfamethoxazole, chloramphenicol, streptomycin, kanamycin, and colistin) were determined using the broth microdilution method according to Clinical and Laboratory Standards Institute (CLSI) M45 guidelines [20]. For antibiotics where species-specific breakpoints are not available for *V. fluvialis*, the breakpoints for *Vibrio* spp. or *Enterobacterales* as recommended by CLSI were applied. Antimicrobial susceptibility testing was performed in cation-adjusted Mueller–Hinton broth (Hopebio, Qingdao, China) supplemented with 2% NaCl, using an inoculum of approximately 5 × 10^5^ CFU/mL. Plates were incubated at 35 °C for 16–20 h. All tests were performed in triplicate. Quality control was performed using *E. coli* ATCC 25922, and all results were within the acceptable ranges specified in CLSI M100 (30th edition) [21].

Conjugation assays were performed as previously described [19], using rifampicin-resistant *E. coli* C600 as the recipient to assess the transferability of both genomic islands. Donor and recipient strains were grown overnight, mixed at a 1:5 donor-to-recipient ratio, and spotted onto nitrocellulose membranes placed on Luria-Bertani (LB) agar (Hopebio, Qingdao, China) plates. After 12–16 h of mating at 37 °C, cells were resuspended, serially diluted, and plated on LB agar containing rifampicin (200 μg/mL) and either meropenem (1 μg/mL) for the detection of SGI1-VfNDM1 transconjugants, or tetracycline (8 μg/mL) for the detection of GI*flu*-1 transconjugants, as the two islands carry different resistance markers. The detection limit of the assay was approximately 10^−9^ transconjugants per recipient. Any colonies growing on the selective plates would have been confirmed by PCR targeting the *bla*_NDM-1_ for SGI1-VfNDM1 and *tet*(A) for GI*flu*-1, and by 16S rRNA gene sequencing to verify the recipient background. However, no transconjugants were obtained for either island under the tested conditions.

### 2.3. Whole-Genome Sequencing and Bioinformatic Analysis

Genomic DNA was extracted, and whole-genome sequencing was performed using the Illumina (short-read) and Oxford Nanopore (long-read) platforms. Illumina sequencing generated 2.1 million paired-end reads (2 × 150 bp). Nanopore sequencing produced 98,000 reads with an N50 of 12.5 kb. The resulting reads were assembled into two complete circular chromosomes (total size 4.9 Mb, 10× coverage) using Unicycler v0.4.8 [22], and genome annotation was carried out using the NCBI Prokaryotic Genome Annotation Pipeline (PGAP, v4.16) and RAST server (https://rast.nmpdr.org/, accessed 10 March 2026). The assembly was validated by read mapping and manual inspection of circularization junctions.

For antimicrobial resistance genes identification, we used ResFinder 4.7 (https://genepi.food.dtu.dk/resfinder, accessed 10 March 2026). Virulence factors were searched against the Virulence Factor Database (http://www.mgc.ac.cn/VFs/, accessed 18 February 2026). Putative genomic islands were identified using IslandViewer 4 (https://www.pathogenomics.sfu.ca/islandviewer/, accessed 16 March 2026), and their genetic structures were visualized using Easyfig v2.2.2 [23]. To delineate the phylogenetic context of strain 10M-VF, all publicly available *V. fluvialis* genome assemblies (*n* = 475) were retrieved from the NCBI GenBank database (accessed 15 May 2026). A preliminary core-genome single nucleotide polymorphism (SNPs) phylogenetic tree was constructed using Roary v3.13.0 [24] and FastTree v2.2 [25] to assess overall phylogenetic diversity and to identify close relatives of 10M-VF. Subsequently, a subset of 31 phylogenetically representative strains was selected from the 475 publicly available genomes, including: (i) all publicly available *bla*_NDM-1_-carrying *V. fluvialis* isolates (*n* = 8); (ii) phylogenetically diverse strains representing the major clades identified in the preliminary 475-strain tree; and (iii) strains with high-quality genome assemblies (complete or near-complete). The selection was performed to ensure representation of diverse geographic and source origins while avoiding over-representation of closely related isolates. Strain 10M-VF was additionally included in the phylogenetic analysis. In total, 9 *bla*_NDM-1_-carrying *V. fluvialis* isolates were analyzed, comprising the 8 publicly available isolates and strain 10M-VF. A final core-genome SNP tree was reconstructed based on this subset for clearer visualization and focused analysis. The tree was visualized and annotated using iTOL v5 (https://itol.embl.de/itol.cgi, accessed 18 May 2026).

## 3. Results

From 38 retail seafood samples, 8 presumptive *Vibrio* colonies were selected after enrichment and selective plating for purification and species identification. Among these, only one isolate was confirmed as carbapenem-resistant *V. fluvialis* (designated 10M-VF), recovered from a mantis shrimp sample. This isolate was identified as *V. fluvialis* by MALDI-TOF MS and corroborated by MLST analysis through the PubMLST database. Antimicrobial susceptibility testing revealed that strain 10M-VF was resistant to meropenem (MIC = 64 μg/mL), imipenem (>128 μg/mL), ceftazidime (>128 μg/mL), and other clinically important antibiotics including ampicillin, ceftriaxone, tetracycline, and sulfamethoxazole, but remained susceptible to ciprofloxacin, gentamicin, and colistin (Table 1). MICs were interpreted using CLSI M45 breakpoints for *Vibrio* spp. where available (e.g., tetracycline, ciprofloxacin, gentamicin, sulfamethoxazole). For antimicrobial agents without *Vibrio*-specific breakpoints in CLSI M45 (including meropenem, imipenem, ceftazidime, ceftriaxone, aztreonam, ampicillin, and colistin), interpretive categories were based on CLSI M100 breakpoints for *Enterobacterales*.

**Figure 1:**
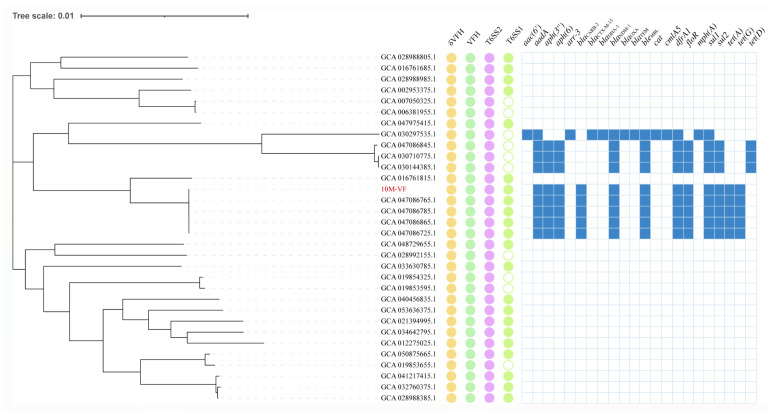
**Genomic sequences analysis of 32 *V. fluvialis* strains.** A core-genome-based phylogenetic tree of these *V. fluvialis* strains is shown. The tree is based on a subset of 32 phylogenetically representative strains selected from a preliminary analysis of all publicly available *V. fluvialis* genomes. Antimicrobial resistance genes are indicated by blue squares. δVFH, VFH, T6SS2 and T6SS1 are indicated by yellow, green, pink, and pale green circles, respectively; solid circles indicate presence and hollow circles indicate absence of the corresponding gene. Detailed strain metadata and the presence of SGI1 or GI*flu*-like islands are provided in Supplementary Table S1.

To elucidate the resistance mechanism, the complete genome of *V. fluvialis* strain 10M-VF was sequenced using a hybrid approach combining Illumina short-read and Oxford Nanopore long-read sequencing, which yielded a high-quality closed genome assembly. The genome consists of two chromosomes, designated chromosome I and chromosome II. Chromosome I is 3,250,262 bp with a GC content of 49.94%, and chromosome II is 1,691,519 bp with a GC content of 50.27%. The complete genome sequences of strain 10M-VF have been deposited under GenBank accession numbers CP118599 (chromosome I) and CP118600 (chromosome II). No plasmid replicon types were identified by PlasmidFinder (v2.1, accessed 12 March 2026) analysis, and no plasmid contigs were detected in the hybrid assembly. The absence of plasmids was further supported by the presence of only two circular chromosomes in the complete genome assembly. A core-genome phylogenetic tree was constructed based on 32 *V. fluvialis* strains. The results showed that all strains encode VFH, δVFH, and T6SS2, whereas 20 strains harbor T6SS1, and only 9 strains carry drug resistance genes. Notably, strain 10M-VF is phylogenetically close to strain GCEnv1 (isolated from soil, GenBank No. GCA_016761815.1) and strain GD23SC5426TM (isolated from shrimp, GenBank No. GCA_047086765.1) (Figure 1), indicating that genetically related strains can be recovered from diverse environmental and food sources.

**Figure 2:**
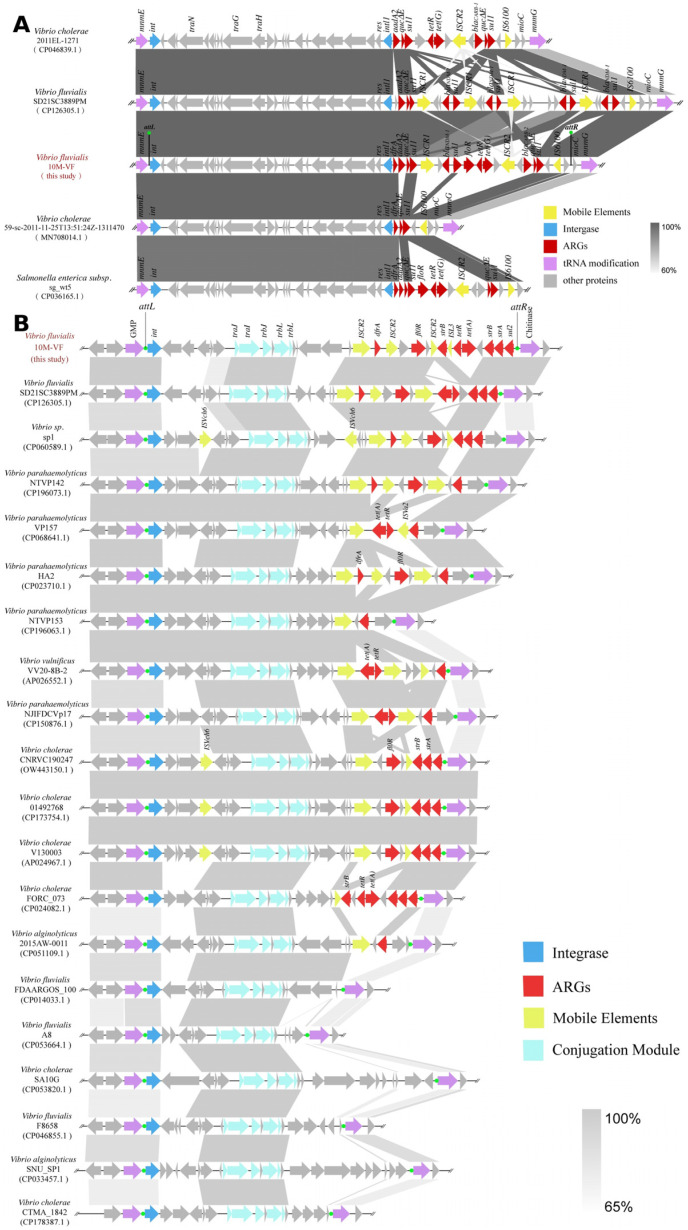
**Comparison of the genomic islands identified in *V. fluvialis* strain 10M-VF with related sequences**. (**A**) Linear comparison of the *bla*_NDM-1_ genetic environment in SGI1-VfNDM1 with those of other SGI1-encoding bacteria. (**B**) Linear comparison of GI*flu*-1 with other GI*flu* variants. Antibiotic resistance genes are indicated in red font. In subgraph B, purple arrows indicate the integration site-flankinggenes (*guaA*, encoding GMP synthase, and a chitinase gene), and grey arrows indicate homologous backbone genes in the 20 compared strains. Green circles indicate *attL* and *attR* recombination sites. AGR, antibiotic resistance gene(s).

Using IslandViewer, we identified a genomic island, designated SGI1-VfNDM1, on chromosome I of strain 10M-VF. By comparing the genome sequence of strain 10M-VF with those of other *V. fluvialis* strains, we found that SGI1-VfNDM1 integrates between *mioC* and *mnmE* genes (Figure 2A). Direct repeats of 18 bp specific recombination sites *attL* (5′-TTCTGTATCGGGAAGTAA-3′) and *attR* (5′-TTCTGTATCGGTAAATAA-3′) were identified. SGI1-VfNDM1 is 47,985 bp with an average GC content of 49.8%. BLASTn analysis showed that the backbone of SGI1-VfNDM1 shares high similarity with SGI1 from other bacterial species (~99% sequence identity and ~90% coverage). However, only SGI1-VfNDM1 harbors the IS*CR1*-*trpF*-*ble*_MBL_-*bla*_NDM-1_ region (Figure 2A). These results indicate that SGI1-VfNDM1 is a new SGI variant, and that IS*CR1* may have mediated the integration of *bla*_NDM-1_ into SGI1-VfNDM1. In addition, a complex class 1 integron, *intI1*-*hp*-*aadA2*-*qacEΔ1*-*sul1*-IS*CR1*-*trpF*-*ble*_MBL_-*bla*_NDM-1_-*qacEΔ1*-*sul1*, was observed in SGI1-VfNDM1. Other resistance genes, including *tet*(G), *qacEΔ1*, *aadA2*, *sul1*, *floR*, and *bla_C_*_ARB-2_, were also detected in SGI1-VfNDM1 (Figure 2A).

Using ResFinder, a cluster of drug resistance genes, including *floR*, *strB*, *tet*(A), *strA*, and *sul2*, in an approximately 31 kb region on chromosome I. These genes lie close to each other but approximately 2470 kb away from SGI1-VfNDM1. It was initially suspected that this region might be a genomic island, but IslandViewer failed to identify it as such. To resolve this, the genome sequence of strain 10M-VF was compared with those of other *V. fluvialis* strains and it was found that this 31 kb region integrates between *guaA* (encoding GMP synthase) and a chitinase gene (Figure 2B). Given the presence of an integrase gene and putative recombination sites *attL* (5′-GAGTGGGAATAAT-3′) and *attR* (5′-GAGTGGGAATAAT-3′), it was concluded that this region is a genomic island, which we designated GI*flu*-1. GI*flu*-1 is 31,603 bp with a GC content of 48.9% and carries a multiple drug region with the structure IS*CR2*-*hp*-*floR*-*hp*-IS*CR2*-*strB*-IS*L3*-*tet*(R)-*tet*(A)-*hp*-*strB*-*strA*-*sul2* (Figure 2B). BLASTn analysis showed that GI*flu*-1 shares less than 69% coverage with any other sequences from *Vibrio* strains deposited in the database. However, a total of 20 sequences (including GI*flu*-1) share a common set of core genes (*int*, *traJ*, *traI*, *trbJ*, etc.), suggesting they are all variants of a common GI*flu* family. The number of resistance genes located on GI*flu* variants varies. In GI*flu* variants, including GI*flu*-1, IS*CR2* is found adjacent to resistance genes (Figure 2B), suggesting that IS*CR2* may contribute to the acquisition of resistance regions within GI*flu* islands.

Conjugation experiments were performed to assess the transferability of SGI1-VfNDM1 and GI*flu*-1. However, no transconjugant was obtained under the tested laboratory conditions, suggesting that these islands may require specific helper plasmids or environmental factors for mobilization.

## 4. Discussion

The identification of two distinct genomic islands in seafood-borne *V. fluvialis* strain 10M-VF highlights the role of this pathogen as a reservoir for multiple resistance determinants. SGI1-VfNDM1 carries *bla*_NDM−1_ within an IS*CR1*-mediated cassette, while GI*flu*-1 represents a novel resistance island carrying *floR*, *strAB*, *tet*(A), and *sul2*. From a veterinary perspective, *V. fluvialis* causes vibriosis in farmed shrimp and fish, leading to significant economic losses [2,3]. Of note, based on analysis of all 475 publicly available genomes, the majority (approximately 85%) of *V. fluvialis* strains do not encode any drug resistance genes, indicating that these traits are not intrinsic but have been acquired by specific lineages. Although carbapenems are not used in aquaculture, the presence of *bla*_NDM-1_ in a seafood-borne isolate raises concerns about the maintenance and potential dissemination of clinically critical resistance genes in the aquatic environment, with possible spillover to human pathogens via the food chain.

Comparative genomic analysis revealed that SGI1-VfNDM1 from strain 10M-VF (mantis shrimp, Nanjing) is nearly identical (94% coverage) to an SGI1 variant, SGI1-4NDM, from a *V. fluvialis* strain isolated from razor clam in Qingdao, China [15]. Despite differences in seafood source and geographic location (approximately 500 km apart), the high sequence conservation across the backbone and the *bla*_NDM-1_-carrying region strongly suggests that this SGI1 variant has disseminated across different *V. fluvialis* strains through horizontal gene transfer. A previous study showed that *bla*_NDM-1_ integrates into SGI-NDM-1 in four *V. cholerae* strains [14]; however, SGI-NDM-1 and SGI1-VfNDM1 share 21% to 78% sequence identity over the regions aligned, indicating that they are distinct. SGI-NDM-1 and SGI1-4NDM also differ in that SGI1-4NDM possesses four copies of *bla*_NDM-1_, while SGI-NDM-1 has only one. In addition, the presence of nearly identical SGI1 islands in *V. fluvialis* and in human-derived isolates such as *Proteus mirabilis* and *Salmonella enterica* [15], indicates that SGI1-like elements may facilitate the exchange of drug resistance genes between clinical, environmental, and aquatic bacteria. The directionality of such transfer cannot be determined from the current data. Moreover, SGI1 elements are known to require helper IncC plasmids for conjugal transfer [14]. No such helper plasmid was detected in strain 10M-VF, which is consistent with the negative conjugation results.

Importantly, GI*flu*-1 is not restricted to strain 10M-VF. Our genomic comparison identified GI*flu*-1 or its close variants in other *Vibrio* species, including *V. parahaemolyticus*, *V. alginolyticus*, *V. cholerae*, *Vibrio* sp., and *V. fluvialis* (Figure 2B). This indicates that GI*flu*-1 has spread across *Vibrio* species rather than being a single-strain event. Notably, IS*CR2* was found only in GI*flu* variants that carry resistance genes, suggesting that this IS*CR2* may be involved in the acquisition of resistance genes into GI*flu* islands, and GI*flu* islands are distinct from the classical GI*sul2*, as they lack the CR2-*sul2* unit that defines GI*sul2* [16,17]. Furthermore, not every GI*flu* variant carries a resistance gene. Given that *Vibrio* species are common pathogens in aquaculture, the dissemination of GI*flu* islands across multiple species underscores the need for ongoing surveillance of this resistance island in the aquatic food chain.

Beyond resistance, strain 10M-VF carries two hemolysins (VFH and δVFH) and two T6SSs. Previous studies have shown that hemolysins play a critical role in *V. fluvialis* pathogenesis through pyroptosis induction and colonic lesions [7,8], while T6SSs are involved in interbacterial competition and host cell infection [9,10]. The convergence of these virulence determinants with two distinct resistance islands in a single seafood-borne isolate is concerning, as it suggests that *V. fluvialis* is equipped with both pathogenic machinery and multidrug resistance. This dual threat heightens the risk for aquatic animal health and complicates potential disease management in aquaculture settings.

This study is based on a single isolate, which limits the generalizability of our conclusions. While the genomic analysis provides valuable insights, the prevalence and epidemiological significance of these resistance islands in *Vibrio* populations require broader surveillance. Second, the use of selective enrichment may have biased the isolation toward meropenem-resistant *Vibrio* strains. Third, no conjugative transfer was detected under the tested conditions, and the mobilizability of the islands remains to be confirmed. Fourth, the virulence potential of the hemolysins and T6SSs has not been functionally tested in this isolate. Finally, the epidemiological significance of these findings is limited by the small sample size and restricted geographic coverage. It should also be noted that CLSI breakpoints for *V. fluvialis* are not fully established. In this study, MICs for tetracycline, ciprofloxacin, gentamicin, and sulfamethoxazole were interpreted using *Vibrio*-specific breakpoints from CLSI M45. For meropenem, imipenem, ceftazidime, ceftriaxone, aztreonam, ampicillin, and colistin, which lack *Vibrio*-specific breakpoints in CLSI M45, interpretive categories were based on CLSI M100 breakpoints for *Enterobacterales*, following the recommendation for infrequently isolated or fastidious bacteria. This approach may not fully reflect the clinical susceptibility of this species, and the resistance interpretations should be interpreted with caution.

In conclusion, this study reports a novel resistance island, GI*flu*-1, in *V. fluvialis* strain 10M-VF that also harbors SGI1-VfNDM1, together with hemolysins and T6SSs. The emergence of a carbapenemase-encoding SGI1 variant in seafood-borne *V. fluvialis* underscores the role of aquatic food products as reservoirs of clinically and veterinary-relevant resistance genes. From a One Health perspective, monitoring the dissemination of such resistance islands is essential to safeguard both aquatic animal and human health, particularly through surveillance of the aquatic food chain and potential gene exchange between environmental and clinical bacteria.

## Figures and Tables

**Table 1:** **Antimicrobial susceptibility testing of *V. fluvialis* strain 10M-VF.** Results are expressed as MIC (μg/mL). Interpretive categories (S, susceptible; R, resistant) are provided where CLSI-validated breakpoints for *Enterobacterales* or *Vibrio* spp. are available.

Antibiotic	MIC (μg/mL)	
	*V. fluvialis* 10M-VF	*E. coli* ATCC 25922
Meropenem	64 (R)	≤0.25
Imipenem	>128 (R)	≤0.25
Ceftazidime	>128 (R)	≤0.25
Ciprofloxacin	1 (S)	≤0.25
Kanamycin	8 (S)	4
Streptomycin	>128 (R)	8
Aztreonam	16 (R)	≤0.25
Ampicillin	>128 (R)	8
Colistin	0.5 (S)	≤0.25
Ceftriaxone	>128 (R)	≤0.25
Tetracycline	16 (R)	2
Gentamicin	2 (S)	≤0.25
Sulfamethoxazole	>128 (R)	16

*E. coli* ATCC 25922 was included as a quality control strain and all quality control values were within CLSI-acceptable ranges.

## Data Availability

The complete genome sequences of strain 10M-VF have been deposited in the NCBI database under BioProject accession No. PRJNA934166. The GenBank accession numbers for the two chromosomes are CP118599 and CP118600.

## Supplementary Materials

The following supporting information can be downloaded at https://www.mdpi.com/article/10.3390/vetsci13070639/s1, Supplementary Table S1. List of 31 publicly available *V. fluvialis* strains and strain 10M-VF used for core-genome phylogenetic analysis, including strain names, GenBank accession numbers, sources, countries, years of isolation, antimicrobial resistance genes, virulence genes, and presence or absence of SGI1 and GI*flu*-like islands.

## References

[1] Tall B.D., Fall S., Pereira M.R., Ramos-Valle M., Curtis S.K., Kothary M.H., Chu D.M.T., Monday S.R., Kornegay L., Donkar T. (2003). Characterization of Vibrio fluvialis-Like Strains Implicated in Limp Lobster Disease. Appl. Environ. Microbiol..

[2] Xiao H., Cui P., Chen J., Meng L., Che X., Ma Z., Wu X., Lu J., Sun S., Zhu G. (2025). Evaluation of the multivalent immune protective effects of the Vibrio fluvialis outer membrane protein VF17320, and its DNA and IgY antibody vaccines in fish. Front. Vet. Sci..

[3] Hossain M., Momen A.M.I., Rahman A., Biswas J., Yasmin M., Nessa J., Ahsan C.R. (2022). Draft-genome analysis provides insights into the virulence properties and genome plasticity of Vibrio fluvialis organisms isolated from shrimp farms and Turag River in Bangladesh. Arch. Microbiol..

[4] Wang L.-P., Zhou S.-X., Wang X., Lu Q.-B., Shi L.-S., Ren X., Zhang H.-Y., Wang Y.-F., Lin S.-H., Zhang C.-H. (2021). Etiological, epidemiological, and clinical features of acute diarrhea in China. Nat. Commun..

[5] Chowdhury G., Pazhani G.P., Dutta D., Guin S., Dutta S., Ghosh S., Izumiya H., Asakura M., Yamasaki S., Takeda Y. (2012). Vibrio fluvialis in patients with diarrhea, Kolkata, India. Emerg. Infect. Dis..

[6] Chowdhury G., Pazhani G.P., Sarkar A., Rajendran K., Mukhopadhyay A.K., Bhattacharya M.K., Ghosh A., Ramamurthy T. (2016). Carbapenem Resistance in Clonally Distinct Clinical Strains of Vibrio fluvialis Isolated from Diarrheal Samples. Emerg. Infect. Dis..

[7] Wang Y., Luo J., Zhao Y., Zhang J., Guan X., Sun L. (2024). Haemolysins are essential to the pathogenicity of deep-sea Vibrio fluvialis. iScience.

[8] Song L., Huang Y., Zhao M., Wang Z., Wang S., Sun H., Kan B., Meng G., Liang W., Ren Z. (2015). A critical role for hemolysin in Vibrio fluvialis-induced IL-1β secretion mediated by the NLRP3 inflammasome in macrophages. Front. Microbiol..

[9] Sun J., Su H., Zhang W., Luo X., Li R., Liu M. (2023). Comparative genomics revealed that Vibrio furnissii and Vibrio fluvialis have mutations in genes related to T6SS1 and T6SS2. Arch. Microbiol..

[10] Huang Y., Han Y., Li Z., Li X., Li Z., Liu P., Liu X., Cheng Q., Fan F., Kan B. (2022). TssI2-TsiI2 of Vibrio fluvialis VflT6SS2 delivers pesticin domain-containing periplasmic toxin and cognate immunity that modulates bacterial competitiveness. Gut Microbes.

[11] Yue C., Gao X., Xiao L., Lu L., Chen J., Lin C., Cai Z., Gao G., Lv L., Liu J.H. (2025). Unexpected high prevalence of carbapenemase-producing bacteria and widely spread of bla(NDM-1)-positive Shewanella spp. in retail shrimp. Int. J. Food Microbiol..

[12] Zheng Z., Xu Y., Ye L., Chan E.W.C., Chen S. (2022). Genomic insights into the emergence and spread of NDM-1-producing Vibrio spp. isolates in China. J. Antimicrob. Chemother..

[13] Chen D., Sun R., Wang J., Chen K., Xie M., Lin Q., Li J., Chen S., Liu X. (2025). Genetic basis of transmission of bla(NDM-1) among foodborne Vibrio parahaemolyticus strains in China. Appl. Microbiol. Biotechnol..

[14] Siebor E., Neuwirth C. (2022). Overview of Salmonella Genomic Island 1-Related Elements Among Gamma-Proteobacteria Reveals Their Wide Distribution Among Environmental Species. Front. Microbiol..

[15] Gao X., Lu L., Yue C., Bai Y., Liu J.H., Lv L. (2023). A Salmonella genomic island 1 (SGI1) carries multiple copies of bla(NDM-1) in Vibrio fluvialis of retail razor clam origin. J. Glob. Antimicrob. Resist..

[16] Partridge S.R., Kwong S.M., Firth N., Jensen S.O. (2018). Mobile Genetic Elements Associated with Antimicrobial Resistance. Clin. Microbiol. Rev..

[17] Zhang G., Cui Q., Li J., Guo R., Leclercq S.O., Du L., Tang N., Song Y., Wang C., Zhao F. (2022). The integrase of genomic island GIsul2 mediates the mobilization of GIsul2 and ISCR-related element CR2-sul2 unit through site-specific recombination. Front. Microbiol..

[18] Cao W., Wang Y., Zheng L., Zhang Z., Liu S., Dong X., Xian W. (2023). Exploitation pattern assessment of the Japanese mantis shrimp (*Oratosquilla oratoria*) resource in the coastal waters of the Shandong Peninsula. Mar. Pollut. Bull..

[19] Wu X., Zhang W., Liu M., Wang Z., Li R. (2025). Genomic Features of Antimicrobial Resistance and Virulence in Multidrug-Resistant Vibrio furnissii. Vet. Sci..

[20] CLSI (2015). Methods for Antimicrobial Dilution and Disk Susceptibility Testing of Infrequently Isolated or Fastidious Bacteria.

[21] CLSI (2020). Performance Standards for Antimicrobial Susceptibility Testing.

[22] Wick R.R., Judd L.M., Gorrie C.L., Holt K.E. (2017). Unicycler: Resolving Bacterial Genome Assemblies from Short and Long Sequencing Reads. PLoS Comput. Biol..

[23] Sullivan M.J., Petty N.K., Beatson S.A. (2011). Easyfig: A Genome Comparison Visualizer. Bioinformatics.

[24] Page A.J., Cummins C.A., Hunt M., Wong V.K., Reuter S., Holden M.T.G., Fookes M., Falush D., Keane J.A., Parkhill J. (2015). Roary: Rapid Large-Scale Prokaryote Pan Genome Analysis. Bioinformatics.

[25] Price M.N., Dehal P.S., Arkin A.P. (2010). FastTree 2—Approximately Maximum-Likelihood Trees for Large Alignments. PLoS ONE.

